# Moral Bioenhancement, Freedom and Reason

**DOI:** 10.1007/s12152-016-9268-5

**Published:** 2016-07-09

**Authors:** Ingmar Persson, Julian Savulescu

**Affiliations:** 1 Philosophy, Linguistics, and the Theory of Science, Göteborgs Universitet, Gothenburg, Sweden; 2 Oxford Uehiro Centre for Practical Ethics, Oxford, England UK; 3 Oxford Uehiro Centre for Practical Ethics, University of Oxford, Suite 8, Littlegate House, St Ebbe’s Street, Oxford, OX1 1PT UK

**Keywords:** Moral bioenhancement, Freedom, Reason, Responsibility, Coercion

## Abstract

In this paper we reply to the most important objections to our advocacy of moral enhancement by biomedical means – moral bioenhancement – that John Harris advances in his new book *How to be Good*. These objections are to effect that such moral enhancement undercuts both moral reasoning and freedom. The latter objection is directed more specifically at what we have called the God Machine, a super-duper computer which predicts our decisions and prevents decisions to perpertrate morally atrocious acts. In reply, we argue first that effective moral bioenhancement presupposes moral reasoning rather than undermines it. Secondly, that the God Machine would leave us with extensive freedom and that the restrictions it imposes on it are morally justified by the prevention of harm to victims.

Over the years John Harris has been one of the most persistent critics of our advocacy of moral bioenhancement (MB), i.e. moral enhancement by biomedical means. Eventually, he has however come around to the view that our disagreement might not be all that extensive: ‘I do not think P&S and I are, all things considered, so far apart… I have no antipathy at all to moral enhancement per se’ ([1]: 140). The disagreement between us seems to boil down to the fact that he sees MB as a greater threat than we do to ‘the survival of either liberty or …the survival of rational strategies for seeing that good triumphs “all things considered”’ ([1]: 139). In other words, his fear is greater that MB will undermine our freedom of choice and action and bypass moral deliberation or reflection on what is best all things considered. We believe that his fears on both counts are overblown, though we do not doubt that MB – like scientific technologies in general – *could* be so administered that his fear turns out to be justified.

Harris does not seem to see the need for motivating emotions alongside moral judgement in order to produce moral action. For instance, if I find myself failing to feel much sympathy with the global poor, he writes that I ‘may need some help in feeling sympathy, but that is a different problem and not … a moral one’ ([1]: 116). ‘I just have to know what’s right and to know that I ought to do what’s right’ ([1]: 116). Enhancement of sympathy, he claims, is ‘ethically otiose’ ([1]: 115). It all sounds as though he is adopting the Socratic view that if you know what is good and right, you will do it.

Nevertheless, he recognizes that there is the problem of weakness of will, which consists precisely in failing to do what one is convinced that one ought to do, but he tries to write it off with metaphors: this ‘problem is not one that requires moral enhancement but something akin to “stiffening the sinews” and “summoning up the blood” ([1]: 116). So far as we can understand, these metaphors must be cashed out in motivational terms, for clearly the problem of weakness of will is a problem of motivation: it occurs because we are not sufficiently motivated to do what we are convinced that we ought to do, for instance, we do not feel sufficient sympathy for the global poor to aid them when we come up against temptations to satisfy our self-regarding desires. To enhance the capacity to feel such sympathy would be to enhance the probability that we do what we believe that we ought. This is an instance of moral enhancement, according to our view.

People who fall victim to weakness of will have *some* motivation to do what they think they ought; their problem is that this motivation is not strong enough to prevail in the face of opposing desires. Some people – at the psychopathic end of the spectrum – apparently feel no sympathy for the plight of others, though they may be intellectually aware that they ought to help. These people stand in even greater need of moral enhancement in our sense: a strengthening of their moral motivation, like their capacity for sympathy or altruism.

Certainly, a strong capacity to feel sympathy is not all it takes to act morally: sympathy is often misdirected, too much focussed on the suffering that is present to our senses at the expense of what is spatio-temporally distant. Sound moral judgement about what is best all things considered is needed as well. But we do not see why a strong capacity for sympathy has to overturn, or bypass moral judgements, as Harris obviously fears; we think that reasoned judgements and an adequate motivational response in light of them are both necessary for moral action. When he describes enhancement of pro-social attitudes like sympathy as ‘designed to bypass reasoning and act directly on attitudes’ ([1]: 81), it seems that he thinks that such enhancement will result in waves of sympathy welling up within us irrespective of whether or not we have reasons to feel sympathy. But that is a misconception: we take enhancement of the capacity to feel sympathy as meaning being capable of feeling more sympathy *in response to reasons for it*. Like Harris ([1]: 116), we recognize that sympathy is necessarily a reaction to some (considered) state of affairs like somebody’s suffering; it is sympathy *for* the suffering. If a biomedical means like a drug were to ‘cloud’ ([1]: 79) or ‘reduce’ (2016) consideration of such states of affairs it could not possibly enhance the capacity for *sympathy*.

To sum up, it seems to us that if Harris were to cash out the metaphors quoted above, he would land in our position that moral enhancement in the sense of enhancement of moral motivation is necessary to make us act morally more often. Enhancement of moral motivation is not inimical to reasoned judgements about morally relevant matters, like suffering, but rather presupposes them. We have hypothesized that biomedical means may be effective in producing such moral enhancement because at least some moral motivation, like the capacity for sympathy or altruism, appears to be biologically based. In support of this hypothesis, we referred to the fact that women in general have this capacity to a greater degree than men. On the basis of this, we suggested that MB could consist in making men in general more like women in general in respect of the capacity for sympathy ([2]: 111–2).

Without acknowledging any awareness of our prior discussion, Harris proposes a ‘radical feminization of men’ ([1]: 85) as a way of achieving moral enhancement. He contends – as we also explicitly did ([2]: 116) – that this would not make men less free because ‘we have no reason to think that human women are less free than their male counterparts’ ([1]: 88). But, amazingly, he concludes that ‘the research evidence’ in favour of his proposal ‘is considerably more robust’ than it is for what we call MB ([1]: 89). However, what he has proposed *is* MB in our sense, albeit of a rather crude sort and an example we cited of it.

To conclude, Harris has come to realize that, with respect to his view is not so different from ours. We believe that if he had read us more closely, he would have seen that the differences are even smaller than he thinks. The disagreement between us is however more substantial with respect to some thought experiments of ours which do not exactly exemplify MB. Here is one of them, based on a famous paper by Harry Frankfurt [3]:

Imagine that you decide to do the morally right thing on the basis of considering reasons for and against, as somebody who is morally responsible is supposed to do. Imagine, however, that there is a freaky mechanism in your brain which would have kicked in if you had been in the process of making, not this decision, but a decision to do something which is morally wrong. The mechanism would then irresistibly have made you decide to do the morally right thing. Hence, you are not free to fall, i.e. you cannot avoid deciding to do the morally right thing. Would the presence of this freaky mechanism then mean that you are not praiseworthy for making the right decision? It is hard to see why it would: after all, the mechanism was never called into operation; it remained idle. In fact, you decided to do the morally right thing for precisely the same reasons as someone whose brain does not feature the freaky mechanism could do, and whose praiseworthiness therefore is not in doubt. It seems plausible to think that what determines whether you are morally responsible and praiseworthy is the *actual* occurrences that led up to your decision, not some merely hypothetical occurrences that could have led up to your decision, but in fact did not. Certainly, owing to the presence of the freaky mechanism, you are not free to decide to act immorally; this is not anything you could do. ([2]: 114–5).

The freaky mechanism does not count as moral enhancement in our vocabulary, since it does not enhance your motivation to do what is morally right. Rather, it deprives you of your freedom – and even ability – to decide to do and do what is wrong.

In reply, Harris writes that this situation is like being in a room that you do not know is locked. Then ‘you certainly would not be free to leave’ ([1]: 93). That is true, but if you think falsely that you could leave, and decide to stay, you decide freely to stay, and stay freely. The fact that you cannot leave the room does not affect your deliberation leading to the decision because you are not conscious of it. Your mental state could be precisely the same as it when you deliberate about whether or not to leave the room believing *truly* that you can leave it. Therefore, your deciding to stay and staying is just as free in the former as in the latter case, in which you really have the option of leaving the room. Harris’s claim that you would not have freedom ‘of choice *and action*’ ([1]: 94) in these circumstances is then false: you not only freely choose to stay, but stay freely.

This might be clearer if we imagine that you have no belief about whether or not the door is locked, but could get up and check. Suppose you decide not to do so because you want to stay in the room in any case. Then you doubtlessly refrained from checking the door freely, since you could have checked it. But this implies that you also stayed in the room freely: it would be peculiar to hold that whether or not you stay freely depends on whether or not the door is locked, which you freely refrain from finding out.

Harris also writes that our view would ‘constitute the definitive refutation of the idea that “ought implies can”’, ([1]: 94) but it would not. We are not committed to the view that it could be the case that you ought to leave the room, though you cannot. We think, however, that it could be true that you ought to stay – which you can do – though the alternative of leaving or not staying is not open to you, and that your staying could be free and responsible under these conditions. In other words, following Frankfurt, we are rejecting the principle of alternate possibilities (PAP), that moral obligation, responsibility and freedom presupposes that you can act otherwise than you in fact do.

We think that our intuitions both about the freaky mechanism and the locked room support this rejection, and Harris presents no reason to think otherwise. He argues:

1. OiC /Ought implies Can/ is incompatible with determinism…
  and
2. Indeterminism implies PAP…
  Then
3. Denying PAP also involves denying OiC. ([1]: 95)

This is curious argument for several reasons. To begin with, it needs to be reformulated to assume the form of a valid argument. We surmise that what Harris has in mind in this. If OiC is incompatible with determinism, it implies indeterminism (since they are exhaustive alternatives). Now

1. OiC implies indeterminism
  and 2 imply
2. OiC implies PAP,

and, by contraposition, this yields (3) if PAP is false (denied), so is OiC.

However, although this reformulated argument is logically valid, it would still be odd for Harris to advance it. For 1 (or 1*) is at odds with what he has earlier declared: ‘I make no assumptions about the viability of a non-deterministic account of free will and for what it’s worth think some version of compatibilism is probably right’ ([1]: 79). But the first premise expresses the *in*compatibilist view that moral obligation is incompatible with determinism (or implies indeterminism). As a compatibilist who, *contra* Frankfurt, endorses 3 (or 3*) he should rather contend that PAP should be interpreted in a way that renders it compatible with determinism, e.g. that the fact we can act otherwise should be understood conditionally, as amounting to the fact that we will act otherwise *if* we decide to do so. If ‘can’ in OiC is read along these lines, 1 is false.

In an adjunct footnote, he concedes accordingly that this argument does not ‘leave open the possibility that a compatibilist reading of OiC is possible’ ([1]: 95n). But why does he suddenly assume incompatibilism when he has earlier told us that he thinks that ‘some version of compatibilism is probably right’? The explanation might be that he assumes that those who defend a compatibilist reading of OiC ‘cannot say that denying PAP logically entails denying OiC’ ([1]: 95n), i.e. that they cannot endorse 3. But that is a confusion: the only thing they cannot consistently say is that denying PAP *incompatibilistically interpreted as involving a contra-causal power to act otherwise* entails denying OiC or, in other words, that OiC entails PAP thus interpreted. They can however consistently assert that there is a compatibilist interpretation of PAP – indicated above – according to which it is entailed by OiC (though they might instead go along with Frankfurt’s denial of this entailment).

All in all, Harris has given us no reason to believe, *contra* Frankfurt, that 3 is true, i.e. that OiC implies PAP, nor made it unequivocally clear whether he thinks that PAP should be understood as being compatible with determinism or as requiring indeterminism. In the interest of clarity, we believe that he should have stuck to his earlier declaration to stay clear of the compatibilism/incompatibilism debate and concentrated on why he thinks it false that the fact that we ought to do an action entails that we have a dual power to act and to refrain from acting, which is the crux in cases like that of the freaky mechanism.

In another publication [4], we utilize the idea of this mechanism to envisage a super-duper computer, the God Machine (GM), which monitors all the mental states of every human person, and is capable of modifying them within nanoseconds, without the subject noticing it. However, it intervenes only to prevent grossly immoral behaviour, like murder, torture and rape. As soon as it registers that someone is about to decide to perform such an act, or immediately after such a decision has been made, it makes the subject change his or her mind, or forget about the whole thing. Consequently, people under the surveillance of GM would not be free to perform such grossly immoral acts intentionally. (We may also imagine that it prevents them from performing such acts unintentionally.) It would however not interfere with deliberation issuing in immoral behaviour on a smaller scale, like petty larceny (let alone deliberation issuing in moral behaviour). The point of this restriction is that GM’s interference with people’s freedom, as such, is something bad which can be justified only if a greater bad – like someone’s being killed, tortured or raped – is prevented.

Harris regards GM as ‘horrific’ ([1]: 92), but he wildly exaggerates its impact on human subjects. He writes: ‘It would… have prevented anyone … from learning from moral mistakes’ ([1]: 99). That is not true: as remarked, it would only prevent people from perpetrating acts that are gravely wrong. They could learn from perpetrating lesser wrongs such as petty larceny and tax evasion. We believe, however, that the results of some immoral acts are so horrific that whatever people can realistically learn from committing them, it is better all things considered if these learning sessions are prevented. Harris is fond of sprinkling his text with quotations from literary authors, but they are all (with the exception of some classical) British: Shakespeare, Milton, Byron, Auden, and Golding. If his choice of literature had been less chauvinistic, it might have included Fyodor Dostoevsky’s *The Brothers Karamazov* and Ivan Karamazov’s diatribe against God. After telling a shocking story of a couple’s systematic abuse of their little daughter, Ivan exclaims to his brother Alyosha:

Do you understand why this infamy must be and is permitted? Without it, I am told, man could not have existed on earth, for he could not have known good and evil. Why should he know that diabolical good and evil when it costs so much? (Bk. V, ch. 4, trans. Constance Garnett).

Perhaps this reading would have made Harris reluctant to say with such assurance that ‘I do not for a moment think “everyone is much better off for the absence of evil”’ ([1]: 105), like the torture of a child, and that GM which prevents such evil is ‘a million times worse than Milton’s God’ ([1]: 105) who permits it for the sake of the goodness of the freedom enjoyed in the execution of this act.

Another exaggeration of his: ‘The proper analogy with the God Machine is selling or giving yourself into slavery’ ([1]: 106). Not so, as should be clear by now, GM only restricts freedom or ability to act to a small extent. Compare with entering into a social contract which imposes laws on us. These laws restrict our freedom to some extent, but in decent states our freedom is still so extensive that we cannot properly be characterized as slaves. To be sure, GM goes further than law and its enforcement: it removes not merely the *freedom* to perform gravely immoral acts, but even the *ability* to do so. Literally, these acts *cannot* be performed when GM is in operation, whereas laws *can* be broken (at the risk of punishment). This might be regarded as a greater interference in the lives of people, but to our minds this is justified by the horrific badness of the acts rendered impossible.

Harris asks: ‘How would the operations of this new megalomaniac /GM/ be regulated, challenged, or even reviewed?’ ([1]: 105) His point is that these procedures are possible in liberal democracies. Although we did not do so in our original article, it is possible to imagine that GM is regularly reviewed by a democratically elected assembly. For instance, there will be a number of types of action which fall on the borderline between what should prevented and what should be permitted. If some of these act-types which are originally permitted start being frequently committed, the case for preventing them grows stronger. But some types of action should be on the black list irrevocably, like killing and torturing innocent and non-consenting people – and other types should be on the white list irrevocably. If members of the assembly were to be in the process of deciding to change the status of the latter two types, GM should be fixed to interfere with such a reclassification. This certainly sets limits to democracy, but remember democracy is not infallible: notoriously, in Germany in 1933 Adolf Hitler was democratically elected.

Clearly, by inventing GM we have managed to provoke Harris who is known as a great provocateur; so presumably more conventional people will find GM even more provocative. Why is that? It might be instructive to compare GM with the Christian God, whose (alleged) existence many seem comfortable with. One feature of GM that people might find disturbing is that it monitors the minds of all people and, thus, has complete knowledge of what goes on in them. This might seem to constitute a gross violation of a right to privacy. But the same would be true of an omniscient God, and yet the complaint that God violates the right to privacy is never heard. In fact, if human persons have a right to privacy, which God would necessarily violate in virtue of being omniscient, it would be inconsistent to assume that God is also perfectly good, or all-good. So, perhaps the right to privacy should be rejected (see [2]: 53–6). But then GM must be acquitted on the score of violation of a putative right to privacy.

In virtue of being omnipotent, God would also be endowed with a power to block decisions to perform morally atrocious actions, like torturing and killing children, but in contrast to GM, God would never make use of this power. Thus, a difference between God and GM would be that God could never be accused of violating any right to autonomy. But a right to autonomy should not be thought to extend so far as to encompass a right to perform morally atrocious acts. Therefore, presumably like Ivan Karamazov, we think that the fact GM prevents such acts counts in its favour. The possible goodness of the freedom their exercise might involve is vanishingly small in comparison to their harmfulness for the victims.

So, we still have not found any reason why people – and there are billions of them – who are comfortable with the belief that something like the Christian God exists should be uncomfortable with the idea of GM. It might be replied that, whereas God is incorruptibly perfectly good, GM might malfunction and wreak havoc on human lives. Thus, we cannot wholly trust GM as we can wholly trust God. But the fact that God allows morally outrageous behaviour to occur, such as the torture of innocent children, is a reason to believe that God is not perfectly good – this is the well-known problem of theodicy. Who knows what a God who allows such atrocious behaviour – not to mention natural catastrophes – could do in the future? After all, if God will never interfere with human freedom, presumably He will not interfere with the rogue psychopath, ideologue or fanatic bent on destroying humanity, a power such a person could easily acquire. Perhaps GM would be after all more reliable. The comparison of GM with the Christian God, then, does not dissolve the mystery of why people are so provoked by the idea of GM.

Now we do not think that GM is likely to become a reality in the foreseeable future, if ever. The point of contemplating a scenario featuring it is to find out whether there is anything that could rationally justify the negative gut reaction most of us are prone to have to it. After all, we think it is a good thing to anticipate criminal actions, e.g. terror attacks, and nip them in the bud, and GM is a particularly effective way of doing that. Nor do we think, contrary to what Harris assumes ([1]: 136), that the development and distribution of biomedical means of moral enhancement are an ‘easy’ way to avert the disasters that threaten humankind. We espouse the way of MB because, though long and difficult, it may be necessary to this end.
